# Primetime for Learning Genes

**DOI:** 10.3390/genes8020069

**Published:** 2017-02-11

**Authors:** Joyce Keifer

**Affiliations:** Neuroscience Group, Division of Basic Biomedical Sciences, University of South Dakota, University of South Dakota Sanford School of Medicine, Vermillion, SD 57069, USA; jkeifer@usd.edu; Tel.: +1-605-658-6355

**Keywords:** bivalent domains, learning genes, *BDNF*, methylation, chromatin, classical conditioning, Tet1

## Abstract

Learning genes in mature neurons are uniquely suited to respond rapidly to specific environmental stimuli. Expression of individual learning genes, therefore, requires regulatory mechanisms that have the flexibility to respond with transcriptional activation or repression to select appropriate physiological and behavioral responses. Among the mechanisms that equip genes to respond adaptively are bivalent domains. These are specific histone modifications localized to gene promoters that are characteristic of both gene activation and repression, and have been studied primarily for developmental genes in embryonic stem cells. In this review, studies of the epigenetic regulation of learning genes in neurons, particularly the brain-derived neurotrophic factor gene (*BDNF*), by methylation/demethylation and chromatin modifications in the context of learning and memory will be highlighted. Because of the unique function of learning genes in the mature brain, it is proposed that bivalent domains are a characteristic feature of the chromatin landscape surrounding their promoters. This allows them to be “poised” for rapid response to activate or repress gene expression depending on environmental stimuli.

## 1. Introduction

Learning and memory requires rapid and coordinated gene expression in response to specific environmental events. Expression of individual learning genes, therefore, requires regulatory mechanisms that have the flexibility to respond with transcriptional activation or repression depending on the type of sensory stimulus, its novelty, or context to select the appropriate physiological and behavioral response. Among these mechanisms are specific epigenetic chromatin architectures surrounding some genomic loci that “poise” them for rapid responses to environmental stimuli. These “bivalent domains” are localized to gene promoters and contain certain histone signatures characteristic of both gene activation and repression [1,2]. This dual functionality allows for a high level of flexibility in gene expression programs, especially those required for learning.

The brain-derived neurotrophic factor (*BDNF*) gene in the mature brain is one such learning gene that is uniquely suited to contain bivalent domains in order to be “poised” for rapid transcriptional activation or repression by environmental stimuli. The *BDNF* gene is a key signal transduction element required for synaptic plasticity and many forms of associative learning [3,4]. Moreover, reduced function of *BDNF* is implicated in Alzheimer′s disease and a host of neurodevelopmental and learning disorders [5,6,7] while elevated expression is a marker for high cognitive function in aging [8]. The *BDNF* gene is a target of several known DNA regulatory mechanisms such as methylation/demethylation and chromatin remodeling. Therefore, *BDNF* is a powerful model gene to study the dynamic epigenetic mechanisms underlying gene expression during learning and memory. In this review, transcriptional mechanisms underlying the regulation of learning genes in the brain, particularly *BDNF*, will be discussed. These include active DNA methylation and demethylation, and regulation of chromatin accessibility by specific histone modifications. Much of the work related to the epigenetic control of gene expression, however, involves developmental genes in embryonic stem cells or neural progenitor cells, and this work, along with studies performed on mature neurons in a learning and memory context, will be discussed. Based on our own research on the regulation of *BDNF* during one form of associative learning, classical conditioning, we hypothesize that learning genes in mature neurons are uniquely suited to contain bivalent domains that “prime” them for rapid transcriptional responses to behaviorally relevant environmental stimuli.

## 2. Active DNA Methylation/Demethylation in Learning and Memory

Active DNA methylation/demethylation and chromatin remodeling are critical to understanding mechanisms of gene expression during learning. Methylation of cytosine (5-methylcytosine; 5mC) is one of the best-studied epigenetic modifications of DNA. It has been primarily characterized at CG dinucleotides but has recently been a focus of studies at non-CG sites, particularly CA [9,10,11]. Patterns of methylation and demethylation across the genome are dynamically regulated by DNA methyltransferases (DNMT) and the methylcytosine dioxygenase 1–3 (Tet1–3) proteins, respectively. The process of demethylation from 5mC to unmethylated C is complex and not fully characterized, but it involves the successive oxidation of 5mC by the Tet1-3 family of dioxygenases [9,12]. These proteins actively convert 5mC to its oxidative derivatives such as 5-hydroxymethylcytosine (5hmC). The 5hmC mark has recently come under intense scrutiny as evidence suggests that it is also a stable epigenetic mark [13,14] that may regulate patterns of gene expression. The presence of 5mC in gene bodies and promoters is widely considered to be an epigenetic mark that acts to suppress transcription whereas Tet-mediated demethylation promotes it [2,9].

Activity-dependent *BDNF* gene expression in early studies was related to decreased CG methylation [15] which correlated with enhanced transcription [16,17]. Methylation induces transcriptional silencing by recruiting methyl-CpG-binding protein 2 (MeCP2) and associated protein complexes to bind tightly to DNA, thereby preventing the access of proteins required for transcription. MeCP2 is a ubiquitous multifunctional regulator of gene expression that binds directly to 5mC at CG and non-CG dinucleotides, particularly methylated CA, and 5hmC [11,14,18,19,20]. In a seminal study from the Sweatt lab, Lubin et al. [21] examined epigenetic *Bdnf* gene regulation in a rat hippocampus subjected to contextual fear conditioning. They found that fear-dependent upregulation of exon IV expression was related to DNA demethylation and histone H3 acetylation in the promoter region. Moreover, inhibition of demethylation with a DNMT blocker interfered with *Bdnf* mRNA expression and conditioning. Supporting the basic model for gene methylation and transcriptional repression, more recent evidence showed that repression of *Bdnf* 48 h after memory consolidation of an inhibitory avoidance task was associated with increased MeCP2 and Sin3A-histone deacetylase 2 (HDAC2) repressor complex binding to *Bdnf* [22]. This occurred after the behavior had been learned and Bdnf protein expression was no longer required. Importantly, it is now recognized that transcriptional activation as well as repression may be associated with MeCP2 binding [20,23,24,25]. Exactly how MeCP2 mediates both gene activation and repression is unknown and a fundamental question in cell biology. One primary line of thinking is that MeCP2 serves as a transcriptional repressor when MeCP2-HDAC complexes with the co-repressor Sin3A [26,27] and binds to DNA, thereby blocking transcription. MeCP2 may also recruit methyltransferase activity directly to histones and repress transcription by modifying chromatin structure [28]. Alternatively, MeCP2 serves as an activator when the MeCP2-Sin3A-HDAC complex dissociates or if MeCP2 interacts directly with the transcription factor cAMP response element-binding protein (CREB) as a co-activator [23]. Supporting a role for activation, genome-wide studies indicate that MeCP2 binds to 5hmC which is enriched in active genes during neuronal differentiation [13] and in terminally differentiated neurons [18,29]. Further, the *Zif268* gene was activated during contextual fear conditioning when bound by MeCP2 [30]. Consistent with this finding, but inconsistent with the model for transcriptional repression by MeCP2, mutant mice in which phosphorylation of MeCP2 at Ser421/424 was abolished, thereby preventing release from DNA, surprisingly, showed better fear and spatial memory, enhanced long-term potentiation (LTP), and increased *Bdnf* expression compared to wild types [31]. These seemingly conflicting results are explained by MeCP2′s dual function as a transcriptional repressor and activator [20,23,24,25].

Tet1 has been implicated in active DNA demethylation and gene regulation during learning and memory but characterization of its activity-dependent actions has so far been limited. Guo et al. [32] reported that *Bdnf* gene promoters in neurons of the adult mouse brain were demethylated by Tet1 in response to electroconvulsive shock treatment to promote gene expression. This response was rapid, occurring within 4 h of treatment. In a different study [33], Tet1-knockout mice in naïve conditions exhibited downregulation of a number of activity-related genes including *Npas4*, *c-Fos* and *Arc* in the cortex and hippocampus. Further scrutiny of the *Npas4* gene indicated that downregulation was associated with hypermethylation of its promoter region and impaired extinction learning during contextual fear conditioning. On the other hand, overexpression of Tet1 in the hippocampus led to a global reduction in 5mC and enhanced 5hmC, and resulted in the selective upregulation of activity-dependent genes such as *Arc*, *Homer1*, and *Bdnf* [34]. Long-term memory of contextual fear was also impaired in these mice, although short-term memory was not. Clearly, these data indicate that Tet1 regulates gene methylation and gene expression in an activity- and learning-dependent manner. Hypermethylation is related to gene suppression while Tet1-mediated demethylation is related to expression. The downstream effects on learning and memory have proven to be complex, however, and likely relate to genome-wide dysregulation of gene expression in studies using knockout or overexpression of Tet1. Finally, while studies of genome methylation patterns and gene expression are establishing a strong link with learning and memory states, will they contribute to understanding the neuropathology of cognitive disorders such as Alzheimer′s and autism spectrum disorders (ASDs)? As technical advances for large-scale, genome-wide analyses of the methylome are developed, significant insights are beginning to emerge. Notably, epigenome-wide examinations of patient populations have provided compelling evidence that altered DNA methylation of specific target genes is associated with Alzheimer′s neuropathology [35,36]. Differentially methylated regions, many hypermethylated, were specifically associated with genes connected to susceptibility for Alzheimer′s. While disorders such as Alzheimer′s likely result from a number of causative factors, understanding the role of gene methylation and its dynamic regulation during learning will contribute an important piece of the puzzle for developing better diagnosis and treatments of neurological disease.

## 3. Histone Modifications in Active and Inactive Genes

The chromatin architecture of genes has a defining role in regulating their expression. Whether or not genes are expressed depends on an open or compact chromatin structure that is regulated in part by post-translational modifications of histones by methylation, acetylation, and other modifications. DNA domains enriched in active histone marks such as trimethylation of histone H3 Lys4 (H3K4me3), H3K36me3, or acetylation of histone H3 (H3ac) are primed for transcription, while those with elevated repressive marks, including H3K27me3 or H3K9me3, are not [2]. Chromatin remodeling is triggered by regulatory proteins that catalyze specific histone modifications. The H3K4me3 mark associated with active transcription is catalyzed in vertebrate cells by SET1A/B and the mixed lineage leukemia (MLL) protein complexes [2,37,38]. Evidence indicates that SET1A/B establishes most genomic H3K4 methylation whereas the actions of MLL are gene-specific and related strongly to gene transcription. On the other hand, the Polycomb-repressive complex 2 (PRC2) has a ubiquitous gene repressor function and catalyzes the repressive H3K27me3 mark. The function of PRC2 has been primarily studied during development in yeast, *Drosophila*, and embryonic stem cells [2,37,38], or in relation to some neurological diseases [39]. While SET1/MLL and PRC2, among others, are likely targets of learning-related mechanisms, little is known about their regulation in the context of learning and memory.

In addition to the DNA methylation discussed above, histone modifications have also been examined in studies of contextual fear conditioning. Binding of the active histone marks H3K4me3 and H3ac to *Bdnf* or *Zif268* promoters in the rat hippocampus were significantly enhanced after conditioning and associated with increased transcription [21,30]. In some cases, these modifications took as little as 30 min. Studies of addictive behavior using chronic drug treatment paradigms have also been used to examine the epigenetic underpinnings of reward responses. Cocaine administration was associated with enhanced transcriptionally active H3ac and reduced repressive H3K9me2 marks in specific target genes that would induce expression and support drug-seeking behavior [40,41]. Further analysis of *Bdnf* promoter histone modifications in a reward region known as the ventral tegmental area (VTA) following chronic morphine administration revealed a significant reduction in H3ac and increased H3K27me3, as well as enhanced binding of the transcriptional repressor PRC2 [42]. These observations corresponded with stalled RNA polymerase II (RNAPII) elongation leading to the suppression of *Bdnf* mRNA. Hence, a series of epigenetic alterations induced by chronic morphine treatment may underlie the reduced levels of *BDNF* gene expression observed in human heroin addicts [42]. Nicotine exposure of the developing brain is known to result in changes in neuronal morphology and persistent alterations in learning. This process was also revealed to have an epigenetic basis [43]. Exposure of fetal mice to nicotine resulted in the elevated expression of several genes, the most prominent of which was *Ash2l* which encodes a core enzyme in the SET1A/B complex that catalyzes H3K4 histone methylation. After nicotine, H3K4me3 was enriched at a number of gene promoters that regulate the development of neuronal dendritic morphology, leading to enhanced dendritic branching and hypersensitive passive avoidance learning. These findings are significant as the H3K4me3 modification is highly represented in active genes and enhances the accessibility of the chromatin architecture to transcription, a modification shown in this study to be directly relevant to behavior following nicotine exposure. Along these lines, a novel viral-mediated system was recently introduced and used to deliver specific histone modifications to the *Cdk5* gene in neurons of adult mice [44]. Deposition of the active H3K9/14ac or repressive H3K9me2 marks increased or attenuated, respectively, cocaine-induced locomotor behavior, demonstrating direct epigenetic effects on behavior.

Unexpectedly, extracellular signal-regulated kinase 1/2 (ERK1/2) has been shown to bind directly to specific DNA sequence motifs and is implicated in regulating chromatin accessibility in embryonic stem cells [45,46]. Evidence indicates that ERK1/2 binds DNA independently of its well-known kinase activity through a distinct DNA-binding domain [46,47]. Genome-wide profiling indicates that ERK1/2-bound promoters exhibit higher levels of the histone modifications H3K4me3, H3K9ac, and H3K27ac found in transcriptionally active genes. Moreover, ERK1/2- and PRC2-targeted developmental genes were found to lack transcription factor II Human (TFIIH), a transcription factor known to phosphorylate RNAPIISer5 required for transcriptional initiation [46]. Tee et al. [46] provided evidence that RNAPII was instead phosphorylated at Ser5 by ERK2 in in vitro studies and in embryonic stem cells. We have also obtained evidence for conditioning-dependent phosphorylation of RNAPIISer5 by ERK1/2 in the mature brain using our model of classical conditioning [48]. Due to its combined actions on histones and RNAPII, ERK1/2 is proposed [46] to bind to promoters of developmental genes and promote a permissive chromatin configuration, making them competent for approach by RNAPII and transcription. This is a novel role for ERK1/2 in gene activation that requires further study.

## 4. Bivalent Domains in Developmental Genes

Bivalent promoter domains contain chromatin features characteristic of both gene activation and repression and have been primarily studied in pluripotent embryonic stem cells during cellular differentiation. Active gene promoters show enrichment at the H3K4me3 mark while repressed promoters are associated with H3K27me3. Promoters of developmental genes having the distinctive H3K4me3 and H3K27me3 features of bivalency are, therefore, considered to be in a transcriptionally “poised” state, ready for rapid gene induction or repression [2]. Bivalency may also be marked by other histone modifications, including the H3K9me3 mark at gene promoters [49]. Moreover, bivalent domains are not limited to promoter regions and have been identified at gene enhancers [50]. Bivalent chromatin domains in promoter regions are typically identified near transcription start sites (TSSs) where they predominate [1,49,51]. Genes having the bivalent signature are associated with a specific form of RNAPII that is bound to DNA but in a paused state [49,52,53]. That is, it contains higher levels of phosphorylated Ser5 residues required for initiation of transcription and lower levels of Ser2 which promotes pre-mRNA elongation [53]. The presence of the H3K27me3 mark is incompatible with active transcriptional elongation, so these genes undergo initiation but not elongation. Bivalency is not limited to pluripotent embryonic stem cells. Cells that have differentiated to neural progenitor cells and become lineage-committed typically show enhanced monovalency toward either H3K4me3 or H3K27me3 [51]. Importantly, a small percent remain bivalent, indicating that differentiated cells contain bivalent genes. Recent evidence indicates that regulation of bivalence may involve repressor mechanisms independent of PRC2 and the H3K27me3 mark [54]. Characterization of RE1 silencing transcription factor (REST)-targeted genes in embryonic stem cells destined to differentiate toward the non-neuronal lineage are poised by H3K4me3 and a repressor complex requiring HDAC activity. Therefore, multiple forms of bivalence and their associated regulatory mechanisms are likely to be present in the genome. Bivalent domains have also been identified in cancer cells but the relationship between hypermethylation generally associated with these genes and gene expression remains to be resolved and is an active area of research [2,55].

In order for genes to be truly bivalent, the H3K4me3 and H3K27me3 marks should be shown to coexist simultaneously on a given gene locus, most convincingly in a single cell. However, an unequivocal demonstration of this has proven difficult to achieve experimentally (see Reference [2] for a discussion). This is because most studies make use of populations of cells to obtain an adequate amount of sample DNA. Inhomogeneous cell populations may, therefore, carry one or the other mark, but not both together. Thus, single-cell approaches currently under development are critical in clarifying the abundance and molecular features of true bivalent domains.

## 5. Learning Genes are “Poised” for Rapid Responses to Environmental Stimuli

Learning genes are uniquely suited to respond rapidly to environmental stimuli. This requirement for behaviorally adaptive learning and memory establishes these genes as likely candidates for regulation by bivalent domains controlling transcriptional activation or repression in mature neurons. One model gene we studied during a form of associative learning, *BDNF*, appears to be regulated in such a manner. *BDNF* gene expression is critical for signaling during many forms of learning and memory and its misregulation is strongly implicated in Alzheimer′s disease and other learning disorders. The vertebrate *BDNF* gene contains a number of non-coding exons, each with its own promoter, which are independently up- or downregulated during learning to control the expression of mature BDNF protein [4,21,30,42,56,57]. Exon-specific regulation of gene expression is thought to be related to brain region, type of neuron, and specific environmental stimulus. Therefore, the *BDNF* gene is exquisitely constructed to respond with a high degree of flexibility in the mature brain.

**Figure 1:**
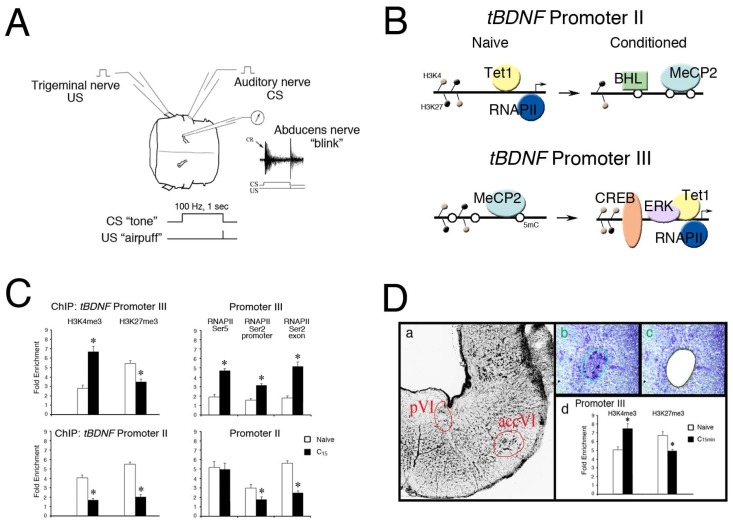
(**A**) Model of in vitro eyeblink classical conditioning. Illustration of the preparation of the pons isolated from the turtle brain showing cranial nerves that are stimulated and recorded. Classical conditioning is generated by paired stimulation of the auditory nerve (the “tone” conditioned stimulus, CS) with the trigeminal nerve (the “airpuff” unconditioned stimulus, US) while recording activity in the ipsilateral abducens nerve which controls blinking in this species. A physiological trace of abducens nerve discharge representative of a neural correlate of a “blink” conditioned response (CR, arrow) is shown. Pairing consists of a 100 Hz, one-second-duration CS to the auditory nerve which precedes a single shock US to the trigeminal nerve. Conditioning is usually recorded after about 50 paired stimuli or in about 1 h; (**B**) Schematic illustration of the epigenetic events controlling *tBDNF* transcriptional regulation during classical conditioning. Promoter II is initially bound by Tet1 and hypomethylated in the naïve state as it is actively transcribed by RNAPII. After 15 min of conditioning, Tet1 dissociates and promoter II is transcriptionally repressed by enhanced methylation (open circles) and binding by MeCP2 and BHLHB2. This is associated with a reduction in both H3K4me3 and H3K27me3 histone modifications. Promoter III is initially methylated and bound by MeCP2 in the naïve state. Conditioning induces demethylation by Tet1, dissociation of MeCP2, and a more open chromatin structure favoring H3K4me3 deposition, allowing access of ERK1/2 and transcriptional activator CREB to initiate *BDNF* transcription and the signaling cascade underlying conditioning; (**C**) ChIP-qPCR assays reveal high levels of both H3K4me3 and H3K27me3 histone modifications for *tBDNF* promoters II and III in naïve preparations. After 15 min of conditioning, H3K4me3 is significantly elevated (*p* < 0.0001) while H3K27me3 is reduced (*p* < 0.01) in *tBDNF* promoter III. The H3K4me3 and H3K27me3 marks for promoter II were both significantly reduced by conditioning (*p* < 0.0001). ChIP of RNAPIISer2 and Ser5 binding to promoter III showed markedly elevated signals after conditioning (*p* < 0.0001) compared to the naïve state, indicating active transcription. For promoter II, RNAPIISer5 binding showed surprisingly high values compared to the naïve state; however, RNAPIISer2 at both promoter and exonic sites was significantly lower than the naïve state (*p* < 0.001), suggesting it is stalled; (**D**) Evidence for *tBDNF* bivalent domains from samples of the abducens motor nuclei obtained by laser microdissection (LMD). Thionin-stained section of the pons (a) showing the two abducens motor nuclei, the principal (pVI) and accessory (accVI), which are spatially separated. The accessory abducens nucleus was demarcated (b) and the cells recovered by LMD (c). Subsequent ChIP-qPCR assays (d) demonstrated bivalent histone modifications in naïve and conditioning-dependent alterations in *tBDNF* promoter III within the region of the CREB binding site. Approximately 20 slices obtained from LMD of the abducens nuclei were pooled [48]. (**A**,**C**), adapted from reference [62].

To investigate activity-dependent epigenetic regulatory mechanisms controlling *BDNF* expression, we studied a neural correlate of eyeblink classical conditioning [58,59,60]. Using an ex vivo preparation from the turtle brain, the cranial nerves are electrically stimulated in place of delivering real stimuli such as an airpuff or tone (Figure 1A). Paired stimulation generates a neural correlate, or “fictive”, physiological discharge representing an eyeblink response that mimics features of conditioning in behaving animals. The advantages of the preparation are that large portions of brain containing intact neural circuits can be maintained in a dish for the extended periods (hours or days) required for studies of learning. Moreover, behaviorally relevant nerve-specific stimulation is used in a conditioning paradigm rather than non-specific stimuli such as glutamate application to induce a neural correlate of learning. This model system allows us to study rapid learning-dependent epigenetic modifications of genes in motor neurons that directly generate the learned behavior.

We have previously characterized portions of the turtle *BDNF* gene (*tBDNF*) and identified three non-coding and one protein-coding exon [57,61]. Regulation of the *tBDNF* gene after conditioning shows selective expression of mRNA transcripts: those encoded by exon I show no change during conditioning, exon II is downregulated, and exon III is substantially upregulated. This latter response triggers the expression of mature BDNF protein which initiates signaling mechanisms underlying conditioned learning in this preparation. Correspondingly, the promoter for exon II is rapidly methylated while, at the same time, promoter III is demethylated [62], as illustrated in Figure 1B, which shows epigenetic modifications of *tBDNF* promoters in the naïve untrained and conditioned states. Enhanced binding of MeCP2 and transcriptional repressor basic helix-loop-helix binding protein 2 (BHLHB2) to *tBDNF* promoter II after conditioning (Figure 1B), demonstrated by chromatin immunoprecipitation and quantitative PCR (ChIP-qPCR), corresponds to the methylation. Likewise, increased binding of Tet1, ERK1/2 and the transcriptional activator CREB to promoter III corresponds to the demethylation. Therefore, there is remarkably coordinated dual control of two *tBDNF* promoters by regulatory proteins during conditioning that are actively regulated by DNA methylation.

Importantly, the histone modifications H3K4me3 and H3K27me3 were both elevated at *tBDNF* promoters II and III in the naïve state, as shown by ChIP-qPCR assays of the promoter regions in Figure 1C [62], suggesting that *tBDNF* contains bivalent domains. After 15 min of conditioning, ChIP showed that *tBDNF* promoter III underwent significantly increased H3K4me3 binding and a corresponding decrease in H3K27me3 compared to naïve, favoring a permissive chromatin structure approachable by DNA-binding proteins. These changes correlate well with the enhanced access of CREB for DNA binding and activation of *tBDNF* III transcripts. This interpretation is corroborated by the increased binding of RNAPIISer5 at the promoter and RNAPIISer2 at both the promoter and exonic regions, verifying productive mRNA transcription (Figure 1C), illustrated in Figure 1B. Nearly the opposite was true for promoter II which was repressed in conditioning. While this promoter is also characterized by relatively high levels of H3K4me3 and H3K27me3 in the naïve state, after conditioning, both marks were significantly reduced. This response may allow access for binding of the transcriptional repressor BHLHB2 [62,63]. Correspondingly, RNAPIISer5 maintained strong binding to promoter II and was poised to initiate transcription but RNAPIISer2 was reduced, indicating it was stalled and unable to engage in productive mRNA elongation. Importantly, Koo et al. [42] also provided evidence for the bivalency of *Bdnf* promoter 2 in neurons of the adult rat. They observed relatively high ChIP signals for H3K4me3, H3ac, and H3K27me3 in control subjects. Following chronic opiate treatment leading to the suppression of *Bdnf*, H3K27me3 was substantially increased while H3ac was reduced, which correlated with stalled RNAPII.

Extending our data supporting *tBDNF* bivalency further, we recently employed the laser microdissection (LMD) technique for sample collection of neurons that specifically undergo learning. The abducens motor nuclei exclusively control eyeblink responses in the turtle (Figure 1Da) [64]. Conditioned responding requires the trafficking of glutamate receptors to synapses in these motor neurons and underlies the learned behavior [60,65]. To provide a more homogeneous cell sample for ChIP-qPCR analysis, abducens motor neurons were collected using LMD (Figure 1D(b,c)). Our findings (Figure 1D(d)) provide further evidence for a bivalent domain within the CREB binding site for *tBDNF* promoter III in naïve preparations that is rapidly converted toward H3K4me3 dominance after conditioning. This modification results in the enhanced accessibility of the chromatin architecture, allowing the binding of CREB for transcriptional activation. These findings provide strong evidence for bivalency at *tBDNF* promoters which undergo rapid modification in response to conditioning. However, even with LMD, cell-to-cell heterogeneity still presents a problem for interpreting bivalent domains. While single-cell epigenetic analysis is still in its infancy, it is currently possible to perform sequential ChIP-sequencing (reChIP-seq) analysis on bulk samples (for example on H3K4me3 and H3K27me3 simultaneously) to lend further support for bivalence in populations of neurons [66].

In addition to the presence of *tBDNF* bivalency, our studies have revealed an essential role for Tet1 and ERK1/2 in classical conditioning and evidence suggests they work together to modify the chromatin landscape [62]. Knockdown of Tet1 using siRNA or inhibition of ERK1/2 by the MEK1/2 antagonist PD0325901 interfered with conditioning-dependent histone modifications, as well as the binding of DNA regulatory proteins (BHLHB2, CREB) and RNAPII. Inhibition of Tet1 or ERK1/2 had a similar effect on their targets, suggesting that the two work together as regulators of gene expression. This is supported by observations that Tet1 and ERK1/2 co-immunoprecipitate and co-occupy the *tBDNF* III CREB binding site during transcriptional activation in conditioning (Figure 1B). Therefore, evidence suggests that *tBDNF* is regulated by Tet1 and ERK1/2 to control chromatin accessibility in a learning-dependent context. One possibility is that Tet1 recruits SET1A/B to establish H3K4me3 histone deposition while ERK1/2 mediates the phosphorylation of RNAPIISer5 to prime the transcriptional machinery.

## 6. Conclusions

*BDNF* is one of many genes essential for learning and memory that must respond with high fidelity to specific sensory stimuli and environmental contexts to generate a behaviorally adaptive learned response. Because of the unique function of learning genes in the mature brain, it is proposed that bivalent domains are a characteristic feature of the chromatin landscape surrounding their promoters. This allows them to be “poised” for rapid response to activate or repress gene expression. In addition to the prototypical *BDNF* gene, there are a number of strong candidate genes for future research to test this hypothesis. These include major players identified in studies of learning and memory such as the *BDNF* transcriptional activator *CREB*, the ubiquitous signaling kinase CaMKII (*CAMK2*), essential for synaptic and structural plasticity [67], the cyclin-dependent kinase 5 (*CDK5*) gene which is implicated in stress responses and cocaine abuse [40,41,44], and the glutamate receptor *GRIA1-4* genes that underlie synaptic potentiation during learning [59,60,65,68]. Other key genes that may contain bivalency in mature neurons are the immediate early genes (IEGs). These genes, which include *c-FOS*, *ZIF268* (*EGR-1*), and *ARC* (*ARG3.1*), have diverse functions and are rapidly expressed in response to neuronal activity [69,70].

Extension of the bivalency concept beyond development to learning genes in mature neurons emphasizes how much more has yet to be understood about epigenetic control of gene expression in a physiological and environmentally relevant context. The importance of this mechanism and its ubiquity across classes of genes remains to be revealed by future work. Such studies will provide insight into how learning genes are misregulated in disease states and whether features of the chromatin architecture for selected genes will prove useful in early detection of cognitive and learning disorders including Alzheimer′s and ASDs.
